# RNA sequencing and iTRAQ proteomic data from an experiment examining the influence of conceptus presence and preovulatory estradiol on endometrial gene transcripts and proteins around maternal recognition of pregnancy in beef cattle

**DOI:** 10.1016/j.dib.2022.108056

**Published:** 2022-03-13

**Authors:** E.J. Northrop-Albrecht, J.J.J. Rich, R.A. Cushman, R. Yao, X. Ge, G.A. Perry

**Affiliations:** a Department of Animal Science, South Dakota State University, Brookings, SD, United States; b USDA, Agricultural Research Service, Roman L. Hruska US Meat Animal Research Center, Clay Center NE, United States; c Department of Mathematics and Statistics, South Dakota State University, Brookings SD, United States

**Keywords:** Conceptus, Preovulatory estradiol, Maternal recognition of pregnancy, Proteomics, Transcriptomics

## Abstract

RNA sequencing reads and isobaric tags for a relative and absolute quantification (iTRAQ)-Based Proteomic Data were used to determine the impact of conceptus presence and preovulatory estradiol concentration on function of the d16 uterus in beef cattle. Conceptuses and endometrial biopsies were collected from the uterine horn ipsilateral to the corpus luteum. Total cellular RNA was extracted from endometrium for RNA sequencing across two lanes of a NovaSeq S2, 2 × 50-bp run. Two independent uterine luminal fluid pools (ULF) were made for each group: highE2/conceptus, highE2/noconceptus, lowE2/conceptus, and lowE2/noconceptus. Peptides were labeled with iTRAQ reagents and analyzed using 2-dimensional liquid chromatography mass spectrometry. Transcript abundances were determined using DESeq2 (FDR <0.05, FC>2). Scaffold Q+ was used to quantitate peptide and protein identifications in ULF. Datasets include uterine transcript and protein abundances among highE2/conceptus vs highE2/noconceptus and lowE2/conceptus vs lowE2/noconceptus groups. This information can be useful for further investigating the role of specific transcripts and proteins in the maintenance of early pregnancy in beef cattle. This dataset is related to the article ‘Influence of conceptus presence and preovulatory estradiol exposure on uterine gene transcripts and proteins around maternal recognition of pregnancy in beef cattle’ by E.J. Northrop-Albrecht, J.J.J. Rich, R.A. Cushman, R. Yao, X. Ge, G.A. Perry. Molecular and Cellular Endocrinology.

**Specifications Table**

**Table:** 

Subject	Animal Science
Specific subject area	The effects of conceptus presence and preovulatory estradiol exposure on the uterine transcriptome and proteome during early pregnancy in cattle
Type of data	Tables
How data were acquired	RNA sequencing, 2D LC-MS/MS iTRAQ
Data format	Raw Filtered Analyzed
Description of data collection	Endometrial biopsies were collected from the ipsilateral uterine horn, and total RNA was extracted. Dual indexed TruSeq stranded mRNA libraries were combined and sequenced on a NovaSeq. Quality reads were mapped to the bovine genome using kallisto, and transcript abundances were identified by DESeq2. Uterine flushes were collected on d16. Two independent pools were made for each group (highE2/conceptus, highE2/noconceptus, lowE2/conceptus, and lowE2/noconceptus). Protein quantification was conducted using a 2D LC-MS/MS based 8plex iTRAQ quantitative method. Samples were pooled, reduced, alkylated by MMTS, digested with Trypsin, and labeled with iTRAQ reagents. Scaffold Q+ was used to quantitate Label Based Quantitation (iTRAQ) peptide and protein identifications.
Data source location	Open Prairie South Dakota State University Brookings, South Dakota United States
Data accessibility	Open Prairie data repository. Article with all data and analysis – 3355 https://openprairie.sdstate.edu/etd/3355/ Supplemental file 1 transcripts from RNA sequencing for the conceptus versus noconceptus – 8.4304 https://openprairie.sdstate.edu/cgi/viewcontent.cgi?filename=8&article=4304&context=etd&type=additional Supplemental file 2 transcripts from RNA sequencing for the highE2/noconceptus versus highE2/conceptus comparison and lowE2/noconceptus versus lowE2/conceptus comparison – 9.4304 https://openprairie.sdstate.edu/cgi/viewcontent.cgi?filename=9&article=4304&context=etd&type=additional
	Supplemental file 3 proteins and their abundances for the highE2/ conceptus versus lowE2/conceptus, highE2/conceptus versus highE2/noconceptus, and the lowE2/conceptus versus lowE2/noconceptus comparisons – 10.4304 https://openprairie.sdstate.edu/cgi/viewcontent.cgi?filename=10&article=4304&context=etd&type=additional Supplemental file 4 protein and peptide summary from the data set generated from ProteinPilot software– 1.4304 https://openprairie.sdstate.edu/cgi/viewcontent.cgi?filename=1&article=4304&context=etd&type=additional Scaffold analysis- highE2conceptus vs lowE2conceptus.csv – 2.4304 https://openprairie.sdstate.edu/cgi/viewcontent.cgi?filename=2&article=4304&context=etd&type=additional Scaffold analysis-highE2conceptus vs highE2noconceptus.csv – 3.4304 https://openprairie.sdstate.edu/cgi/viewcontent.cgi?filename=3&article=4304&context=etd&type=additional Scaffold analysis-lowE2conceptus vs lowE2noconceptus.csv – 4.4304 https://openprairie.sdstate.edu/cgi/viewcontent.cgi?filename=4&article=4304&context=etd&type=additional Mass spec raw file.sf3 8 plex- 6.4304 https://openprairie.sdstate.edu/cgi/viewcontent.cgi?filename=6&article=4304&context=etd&type=additional Mass spec raw file LTQ – 7.4304 https://openprairie.sdstate.edu/cgi/viewcontent.cgi?filename=7&article=4304&context=etd&type=additional
Related research article	E.J. Northrop-Albrecht, J.J. Rich, R.A. Cushman, R. Yao, X. Ge, G.A. Perry, Influence of conceptus presence and preovulatory estradiol exposure on uterine gene transcripts and proteins around maternal recognition of pregnancy in beef cattle. Mol. Cell. Endocrinol. 540 (2021) 111,508. DOI: 10.1016/j.mce.2021.111508w[1]

**Value of the Data**

• This RNA sequencing and mass spectrometry data allows for the identification of transcripts and proteins in the uterus involved in the maintenance of early pregnancy.
• Researchers can use this genomic and proteomic information to further examine uterine environment during pregnancy and conceptus development.
• This genomic and protein data from the paired comparisons are an important resource that can be used as a guide in the selection of genes/proteins for further hypothesis driven studies on reproductive success in cattle.

## 1 Data Description

RNA sequencing reads and iTRAQ-Based Proteomic Data was analyzed in order to determine the impact of conceptus presence and preovulatory estradiol on the d16 bovine uterine transcriptome and proteome. Total cellular RNA was sequenced across two lanes of a NovaSeq S2, 2 × 50-bp run using indexed TruSeq stranded mRNA libraries. Approximately 24 M reads were evaluated for quality for each sample. Reads meeting quality threshold were mapped using kallisto to the bovine reference genome (ARS-UCD1.2) and analyzed for differential expression using the bioconductor package, DESeq2. **Supplemental file 1** shows the complete list of transcripts from RNA sequencing for the conceptus versus noconceptus comparison. **Supplemental file 2** shows a complete list of transcripts from RNA sequencing for the highE2/noconceptus versus highE2/conceptus comparison and lowE2/noconceptus versus lowE2/conceptus comparison.

Two independent uterine luminal fluid pools (ULF) were made for each grouping with each animal in the pool contributing equally to their appropriate pool. Pool groupings were highE2/conceptus, highE2/noconceptus, lowE2/conceptus, and lowE2/noconceptus. Samples were reduced, alkylated by MMTS, and digested with trypsin. Peptides within the samples were then labeled with iTRAQ reagents (8plex). Proteins and peptides were identified using Scaffold Q+. **Supplemental file 3** shows the complete list of proteins and their abundances for the highE2/ conceptus versus lowE2/conceptus, highE2/conceptus versus highE2/noconceptus, and the lowE2/conceptus versus lowE2/noconceptus comparisons. **Supplemental file 4** shows the protein and peptide summary from the data set generated from ProteinPilot software.

## 2 Experimental Design, Materials and Methods

### 2.1 Animals’ experimental design

Angus crossed beef cows/heifers (*n* = 29) were synchronized using the CO-Synch protocol. GnRH (100 µg Factrel i.m.; Pfizer Animal Health, Madison, NJ) was administered on day −9, PGF2 alpha (PG; 25 mg Lutalyse i.m.; Pfizer Animal Health) was administered on day −2, on day 0, cows were artificially inseminated (AI) and concurently administered GnRH (100 µg Factrel i.m.). Estrus activity was monitored by visual observation and EstroTect patches (Western Point, Inc., Apple Valley, MN) from day 0 through day 3. All animals used in the study ovulated following AI. Based on preovulatory estradiol concentrations (>4.9 or <4.9 pg/mL; [2,3]) and expression of estrus animals were grouped into either highE2 or lowE2.

### 2.2 Conceptus recovery

On day 16, a subset of animals had their uteri flushed according to standard non-surgical industry practices with a modified Foley catheter. The uterine horn ipsilateral to the corpus luteum was flushed with 100 mL of flush media. All other animals in the study were slaughtered on d16 and their reproductive tracts were flushed post slaughter with 20 mL or 30 mL of flush media by suturing a plastic tube in the uterine ipsilateral horn tip and clamping off the contralateral horn. Uterine flush fluid was collected and examined to determine if a conceptus was present.

### 2.3 Endometrium collection, RNA extraction, and RNA sequencing

Endometrium samples were collected from animals flushed non-surgically at the curvature of the ipsilateral uterine horn using a Jackson Uterine Biopsy instrument (Universal Surgical Instruments and Better Surgical Instrumentation). Samples were not collected on heifers. Among animals that were slaughtered, endometrial samples were collected from the uterine horn ipsilateral to the corpus luteum by cutting midway down the horn anterior to the bifurcation. Qiagen RNeasy Plus Mini Kits (Austin, TX) were used to extract total cellular RNA. Concentration and integrity were then determined using a spectrophotometer and Agilent RNA Screen Tape System. Samples with a RIN > 7 were submitted for RNA sequencing. Dual indexed TruSeq stranded mRNA libraries (*n* = 29; quality scores equal or greater than 30) were combined and sequenced across two lanes using a NovaSeq S2, 2 × 50-bp run. Approximately 24 M reads for each sample were evaluated for quality. Reads meeting quality threshold were mapped using kallisto to the bovine reference genome (ARS-UCD1.2), and analyzed for differential expression using the bioconductor package, DESeq2.

### 2.4 In-Solution digestion and iTRAQ labeling

Two independent ULF pools were created for each grouping (highE2/conceptus, highE2/noconceptus, lowE2/conceptus, and lowE2/noconceptus). Each animal contributed equally to their appropriate pool. Samples were submitted to the Center for Mass Spectrometry and Proteomics at the University of Minnesota. For each pool, 40 ug was combined with three times the volume of protein denaturation buffer [7 M urea, 2 M thiourea, 0.4 M triethylammonium bicarbonate (TEAB) pH 8.5, 20% methanol and 4 mM tris(2-carboxyethyl)phosphine (TCEP)]. The samples were vortexed briefly, and incubated at 37 °C for 1 h. After incubation, 200 mM methyl methanethiosulfonate (MMTS) was added (8 mM final concentration MMTS), samples were briefly vortexed, and incubated for 15 min at room temperature. All samples were diluted fourfold with ultra-pure water, trypsin (Promega, Madison, WI) was added (1:40 ratio of trypsin to total protein), and they were incubated overnight for 16 h at 37 °C. They were then placed at −80 °C for 30 min, and dried *in vacuo*. Each sample was then cleaned with a four mL Extract Clean™ C18 SPE cartridge from Grace-Davidson (Deerfield, IL). Eluates were dried *in vacuo* and resuspended in dissolution buffer from the iTRAQ kit so the concentration was 2 ug/ul. For each iTRAQ 8-plex label, a 20 ug aliquot of sample was transferred to a new tube and labeled according to the manufacturer's protocol. The samples were then combined into a new tube and dried *in vacuo*. The multiplexed sample was then cleaned with a four mL Extract Clean™ C18 SPE cartridge, and the eluate was dried in a Speedvac vacuum concentrator.

### 2.5 Offline fractionation and mass spectrometry

The iTRAQ 8-plex sample was resuspended in Buffer A (10 mM ammonium formate pH 10 in 98:2 water:acetonitrile) and fractionated offline by high pH C18 RP chromatography [4]. A MAGIC 2002 HPLC (Michrom BioResources, Inc., Auburn, CA) was used with a C18 Gemini-NX column, 150 mm x 2 mm i.d., 5 um particle, 110 Å pore size (Phenomenex, Torrence, CA)with Buffer A being 10 mM ammonium formate pH 10 in 98:2 water:acetonitrile, and Buffer B was 10 mM ammonium formate pH 10 in 10:90 water:acetonitrile. The flow rate was 100 ul/min with a gradient from 5 to 35% Buffer B over 60 min, followed by 35–60% over 5 min. Fractions were collected every 2 min and UV absorbances were monitored at 215 nm and 280 nm. Peptide containing fractions were divided into two equal numbered groups (“early” and “late”). The first “early” fraction was concatenated with the first “late” fraction, which continued. Concatenated fractions were dried down prior to mass spectrometry.

### 2.6 Liquid chromatography and mass spectrometry

Approximately, 400 ng of reconstituted peptide was analyzed with direct column load by capillary LC-MS on an Orbitrap Velos (ThermoFisher, Walthan MA) MS system in data dependent acquisition (DDA) mode with HCD (higher-energy collision induced dissociation) fragmentation mode as previously described [5]. Minor modifications to the capillary LC and MS parameters were: the capillary column diameter was 100 um, the gradient elution profile was of 9–35% buffer B over 65 min at 330 nanoliters/min, HCD activation time was 20 ms; lock mass was off; dynamic exclusion settings were: repeat count = 1, exclusion list size = 500, exclusion duration = 30 s, exclusion mass width (high and low) was 15 ppm and early expiration was disabled.

### 2.7 Data analyses

Only reads of high quality were mapped to the bovine reference genome (ARS-UCD1.2) using kallisto [6]. A negative binominal distribution was fit to all genes after they were filtered to a cutoff of 0.5 counts per million reads. The bioconductor package, DESeq2, was used to analyze differential expression, and differential expression was defined as having a false discovery rate (FDR) <0.05 and a fold change of greater than 2.We analyzed peptide tandem mass spectra with Sequest (XCorr only) in Proteome Discoverer 2.1.0.81 (Thermo Fisher Scientific, Waltham, MA). We used the UniProt (http://www.uniprot.org/) bovine protein database from March 3, 2013 (canonical and isoforms version) combined with the contaminants database (http://www.thegpm.org/cRAP/index.html) for a total of 24,234 protein sequences as the reference database. The protein sequence database contained both reviewed and unreviewed entries. The Sequest database search parameters included: trypsin enzyme with 1 missed cleave sites, fragment ion mass tolerance of 0.1 Da, precursor ion tolerance 50 ppm, methylthio cysteine as a fixed modification. The variable modifications were pyroglutamic acid from glutamine, deamidation of asparagine, oxidation of methionine, N-terminal protein acetylation and iTRAQ 8plex for lysine and peptide N-terminus. The database search results from Proteome Discoverer were processed in Scaffold Q+ (version Scaffold_4.8.4, Proteome Software Inc., Portland, OR) in order to analyze pairwise comparisons. Peptides were identified when the Scaffold Local FDR algorithm was greater than 95.0%, and when at least two identified peptides with greater than 99.0% probability were contained in a protein. The Protein Prophet algorithm was used to assign protein probabilities. When similar peptides were contained in a protein but could not be differentiated based on mass spectrometry analysis principles of parsimony grouping was performed. As described in Statistical Analysis of Relative Labeled Mass Spectrometry Data from Complex Samples, normalization was performed iterative (across samples and spectra) using ANOVA [7]. Sample medians were used for averaging. Log-transformation was performed on all spectra, pruned of matched multiple proteins, and weighted by adaptive intensity weighting logarithm. False discovery rates were adjusted using Benjamini-Hochberg procedure (*P* < 0.05) and significance was based on permutation tests.

## Ethics Statement

All procedures performed in this project were approved by the South Dakota State University Institutional Animal Care and Use Committee and conformed to the Guide for the Care and Use of Agriculture Animals in Research and Teaching.

## CRediT authorship contribution statement

**E.J. Northrop-Albrecht:** Methodology, Formal analysis, Writing – original draft. **J.J.J. Rich:** Methodology, Writing – review & editing. **R.A. Cushman:** Methodology, Writing – review & editing, Formal analysis. **R. Yao:** Formal analysis. **X. Ge:** Formal analysis. **G.A. Perry:** Funding acquisition, Conceptualization, Supervision, Writing – review & editing.

## Declaration of Competing Interest

The authors declare that they have no known competing financial interests or personal relationships which have or could be perceived to have influenced the work reported in this article.

## Data Availability

Raw files from LCMS/MS (Original data) (Open Prairie).
